# Supplementary material for: Identification of photoperiod-induced specific miRNAs in the adrenal glands of Sunite sheep (Ovis aries)
Source: Front Vet Sci. 2022 Jul 22;9:888207. doi: 10.3389/fvets.2022.888207 (PMC9354845; doi:10.3389/fvets.2022.888207)
Supplement: Supplementary file 1 [file Data_Sheet_1.ZIP › Supplementary file/miRDeep2 file.zip!/miRDeep2 file/Know_Structure/SP42A2/oar-mir-329a.pdf]

miRBase precursor	: oar-mir-329a
Total read count	: 74
oar-miR-329a-5p read count	: 3
oar-miR-329a-3p read count	: 70
remaining reads	: 1

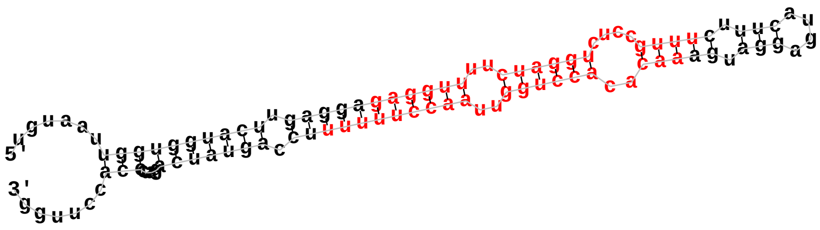

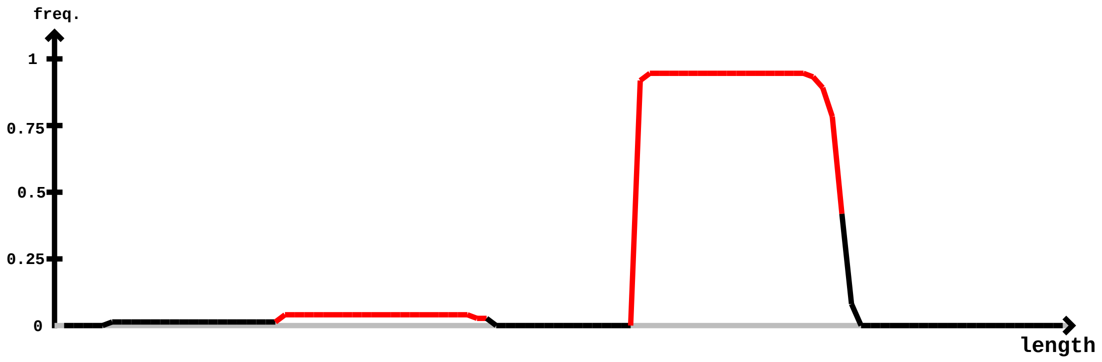

oar-miR-329a-5p

oar-miR-329a-3p

```
5'- uguaauuggugguacuugaggagagguuuucuaggucuccguuucuuucaugaggaugaaacacaccugguuaaccuuuuuuccaguaucagaucccaccuugg   -3'    exp
    .......((((((((.((((((((((..((((((....(((((.(((....))).)))))..))))))..))))))))))).)))).......))))).... reads      mm         sample
    ....auuggugguacuugagga..................................................................................  1          0          seq
    ......................gagguuuucuaggucuccgu..............................................................  1          0          seq
    ......................gagguuuucuaggucuccguuu............................................................  2          0          seq
    ...........................................................aacacaccugguuaaccu...........................  1          0          seq
    ...........................................................aacacaccugguuaaccuu..........................  3          0          seq
    ...........................................................aacacaccugguuaaccuuu.........................  7          0          seq
    ...........................................................Cacacaccugguuaaccuuu.........................  1          1          seq
    ...........................................................aacacaccugguuaaccuuuu........................  27         0          seq
    ...........................................................aacacaccugguuaaccuuuuu.......................  24         0          seq
    ...........................................................aacacaccugguuaaccuuuuuu......................  5          0          seq
    ............................................................acacaccugguuaaccuuuuu.......................  1          0          seq
    ............................................................acacaccugguuaaccuuuuuu......................  1          0          seq
```
